# Sarcoidosis: Sex-Dependent Variations in Presentation and Management

**DOI:** 10.1155/2014/236905

**Published:** 2014-06-02

**Authors:** Andrea D. Birnbaum, Lana M. Rifkin

**Affiliations:** Department of Ophthalmology, Northwestern University Feinberg School of Medicine, 645 North Michigan Avenue, Suite 440, Chicago, IL 60611, USA

## Abstract

Sarcoidosis is an inflammatory disease with a wide range of clinical presentations. The manifestations and prognosis in sarcoidosis are dependent upon not only organ involvement but also age and sex. The purpose of this review is to describe the systemic and ocular manifestations of sarcoidosis with a specific focus on sex-dependent difference in presentation and management. Sarcoidosis is more common in women, particularly in patients who present after age of 50 years. Women with sarcoidosis are more likely to develop cystoid macular edema and the mortality rate is higher than that of men.

## 1. Introduction

Sarcoidosis is a systemic inflammatory disease of unknown etiology that can target almost any organ of the body. The most commonly involved organs include the lungs, lymph nodes, skin, and eyes [1]. The clinical presentation and disease course can be extremely variable depending upon the patient population and organ involvement. More than half of patients diagnosed with sarcoidosis experience a limited disease course and remission within 3 years [2]. Up to a third of patients develop chronic disease and require long-term therapy [3]. Patients with severe pulmonary disease, cardiac disease, or neurosarcoidosis have an increased mortality risk [4] and often require aggressive immunosuppression.

Patients suspected of having sarcoidosis may undergo a battery of diagnostic tests that are not specific for sarcoidosis, but rather markers of granulomatous inflammation. Angiotensin converting enzyme (ACE) may be elevated in 60–70% of patients with sarcoidosis [1]. The serum levels correlate with the disease burden [5]. Lysozyme is another marker of disease activity that, when elevated, might suggest a diagnosis of sarcoidosis. It has a higher sensitivity but lower specificity than ACE for systemic sarcoidosis [6]. Both ACE and lysozyme levels can be elevated in cerebrospinal fluid in neurosarcoidosis [7]. Hypercalcemia and hypercalciuria are present in 10% and 30% of patients, respectively, and are thought to be associated with increased calcium absorption [8]. Vitamin D dysregulation with hypervitaminosis has also been measured in patients with active disease [9]. Because the lungs are the most common site of involvement, chest X-ray or computerized tomography is performed on most patients to aid in diagnosis and identify possible sites for biopsy [10]. Positron emission tomography (PET) is a more comprehensive study that helps clinicians to understand the extent of organ involvement and to identify possible sites for biopsy [11].

While various diagnostic tests can support a diagnosis, none are confirmatory, and, thus, sarcoidosis is considered a diagnosis of exclusion. Experts agree that three criteria must be met prior to assigning a diagnosis of sarcoidosis: (1) clinical or radiologic findings consistent with sarcoidosis, such as pulmonary disease, uveitis, mediastinal hilar lymphadenopathy, or erythema nodosum; (2) tissue biopsy with histologic evidence of noncaseating granulomas; (3) absence of other causes of granulomatous disease [12]. A diagnosis of sarcoidosis can be made based solely on clinical features in select cases, such as Löfgren's syndrome. The classic triad of Löfgren's syndrome is fever, bilateral hilar lymphadenopathy, and polyarthralgias [13]; erythema nodosum is now considered additional diagnostic criterion [14]. In these patients, a diagnosis of sarcoidosis can be made in lieu of biopsy [12–14]. In patients who are unable to undergo biopsy or who do not have appropriate sites for biopsy, the diagnosis is frequently delayed [15].

Ocular involvement occurs in 11–83% of cases of sarcoidosis patients and can lead to significant morbidity [16–18]. The diagnosis of sarcoidosis in patients with ocular disease can be complicated, as intraocular biopsy is not commonly performed for this disease. One retrospective review of patients with biopsy-proven sarcoidosis and uveitis suggested that although the sensitivities of ACE and lysozyme are low in isolation, when both tests are used in combination with chest X-ray, over 80% of patients had at least one marker suggestive of sarcoidosis. The rate increases to over 90% when ACE and lysozyme are combined with a chest CT [19].

Initial management of patients with systemic sarcoidosis is challenging because evidence exists that the use of systemic corticosteroids actually increases the likelihood of relapse [20]. In patients with chronic systemic disease who are effectively treated with corticosteroids, as many as 74% may relapse within 1 month of stopping the medication [2]. In contrast, only approximately 14% of patients who go into spontaneous remission without treatment relapse [2]. In the case of active ocular disease, patients are treated if symptomatic or if they develop vision threatening sequelae of ocular inflammation, such as cystoid macular edema or retinal ischemia. Mild intermediate uveitis does not always require treatment.

## 2. Differences in Incidence and Prevalence

Understanding the epidemiology of sarcoidosis is complicated by the variability in presentation and diagnostic criteria and the disease is certainly underdiagnosed. As an example, one recent study reported sarcoidosis in 22% of patients with mediastinal incidentalomas identified on chest CT [21].

Sarcoidosis presents most often in young adults under the age of 50, with the highest incidence reported in patients between 20 and 39 years of age [22]. Countries with a high prevalence of sarcoidosis include Scandinavian countries [23, 24], which include predominantly white patients, and the United States, where black patients are more often affected [22, 25] and are diagnosed almost 10 years earlier than whites [26]. Black Americans are also more likely to have extrapulmonary disease and a chronic disease course [1, 26].

As with many inflammatory diseases, sarcoidosis affects women more than men. Three population-based studies performed in three different countries have produced similar findings. In Japan, the rates were 1.2 versus 1.4 per 100,000 in males versus females [27] and, in the United States, the rates were 5.9 versus 6.3 cases per 100,000 person-years in males versus females [28]. A much higher incidence was measured in Sweden, and again the rate was higher in females (21.7 per 100,000 person-years) versus males (16.5 per 100,000 person-years) [29]. A more recent study of patients evaluated at a tertiary referral center found that 65.5% were female. Interestingly, men developed symptoms of sarcoidosis and were diagnosed approximately 2 years earlier than women [26].

The increased incidence of sarcoidosis in females has led some to hypothesize that hormones may be a compounding factor. The Black Women's Health Study (BWHS) followed black women between 1995 and 2009 and determined that factors not associated with an increase in incidence of sarcoidosis included age at menarche, age at menopause, and parity. A reduced incidence of sarcoidosis was noted with a later age at first birth, and a weak association was suggested between recent birth and reduced incidence of sarcoidosis [30], although other studies have shown an increase in disease onset in the first postpartum year [31]. One Danish cohort study reported a positive association between the number of children and risk of erythema nodosum [31]. The relative risk of a hospital contact for sarcoidosis was 1.61 in patients with 4 or more children versus women without children [31].

A second trend is the recognized second peak of sarcoidosis diagnosed in patients over 50 years of age [25, 27, 32]. Late-onset sarcoidosis is more common in women than men, which may factor into the increased mean age of diagnosis reported in women versus men [26]. One study reported a rate of 70.3% females in a study of patients with systemic sarcoidosis diagnosed at 70 years of age or older [33]. A second study compared patients with sarcoidosis who were diagnosed at 65 years of age or older with younger patients. In this study, 83.3% of patients in the older cohort were female versus 50% in the younger group (*P* = 0.003). Uveitis was more common in the elderly group versus the younger group (33.3% versus 8.6%, resp.; *P* = 0.002), and 80% of the patients with uveitis in the study were female [34].

The relapse rate of systemic sarcoidosis may also be influenced by sex. White males reportedly have a higher relapse rate of sarcoidosis than white females and African Americans of either gender [2]. In this same study, patients with musculoskeletal sarcoidosis as their presenting disease were more likely to develop recurrence, while patients with asymptomatic disease identified on chest imaging were most likely to remain in remission [2].

## 3. Gender Differences in Clinical Manifestations

### 3.1. Systemic Manifestations

The array of clinical manifestations associated with sarcoidosis is quite varied and includes both constitutional and organ-specific presentations. In any patient with suspected sarcoidosis, a complete review of systems is essential to help characterize the systemic disease and direct diagnostic testing and management. Generalized symptoms of sarcoidosis include fatigue, night sweats, weight loss, arthralgia, and exercise intolerance [35]. Gender differences in complaints of fatigue [36], anxiety, or depression [37] have been measured in patients with sarcoidosis, but they ultimately paralleled similar studies of the general population. Therefore, the authors concluded that sex differences are not specific to sarcoidosis.

Organ-specific involvement is easier to characterize and several sex differences have been identified in this regard. Males tend to have higher rates of pulmonary and cardiac involvement, while females are more prone to peripheral lymph node, skin, eye, and liver disease [26, 38, 39]. The most common cutaneous manifestation is erythema nodosum (EN), which consists of red, tender bumps or nodules on the anterior aspect of the leg. Lupus pernio, a chronic manifestation of sarcoidosis, is characterized by indurated plaques and discoloration of the skin. It often presents on the nose, cheeks, lips, and ears, most commonly in women of African descent. A prospective study of patients with newly diagnosed sarcoidosis conducted in the United States reported that women are more likely to have EN than men, although no significant variation was measured for other cutaneous manifestations [25]. EN can be associated with joint swelling [40], and men with Löfgren's syndrome are more likely to experience periarticular inflammation or arthritis of the ankles [41].

Endocrine abnormalities are common and possibly underreported in sarcoidosis. Hypercalciuria and hyperprolactinemia have been reported in up to 30% of patients with sarcoidosis [9, 42]. Abnormalities of calcium metabolism are more common in men [25]. Clinical manifestations of hyperprolactinemia are certainly sex-dependent, with men experiencing decreased libido, impotence, and gynecomastia and women complaining of secondary amenorrhea, galactorrhea, or decreased libido [42]. Thyroid disorders have also been described in patients with sarcoidosis. Patients with abnormal 67Ga-citrate uptake at initial presentation of sarcoidosis, particularly females with anti-thyroid peroxidase antibodies, are at risk of aggressive autoimmune thyroiditis and hypothyroidism and should be carefully monitored [43].

Genitourinary sarcoidosis will obviously differ between men and women. Among men, clinical signs include testicular swelling and a painless mass in the scrotum [44], while women may experience granulomatous inflammation of the uterus, abnormal bleeding, or erosion of the cervix [45]. Genitourinary sarcoidosis affects less than 0.2% of men with sarcoidosis, although evidence of subclinical involvement is present in up to 5% of patients [44]. Black men are affected at a frequency 10 times greater than white men.

### 3.2. Ocular Inflammation

Sarcoidosis can involve almost any ocular structure, including the globe, orbit, and adnexa. As with systemic sarcoidosis, ocular manifestations of the disease are more common in women [46–48]. In African Americans, the combination of ocular and neurologic disease may be evident [26]. Anterior uveitis is the most common ocular manifestation of sarcoidosis [49] and posterior segment involvement carries a worse visual prognosis [50]. Sex-specific differences in posterior segment disease have been described, with a higher rate of cystoid macular edema (CME) and worse visual acuity in women [51, 52] as well as a trend toward a higher rate of periphlebitis vitreous opacity in men [52].

Several studies have demonstrated a second peak of uveitis in patients over the age of 50, and the vast majority of patients in this subset are female [19, 49, 50]. One consideration in any patient over 50 who presents with newly diagnosed uveitis must be malignancy. In one series, nine patients over the age of 50 were initially diagnosed with primary intraocular lymphoma, but the diagnosis was later determined to be ocular sarcoidosis. Seven of the patients had multifocal choroiditis and six had cystoid macular edema, both of which are rare in lymphoma [53]. This highlights two considerations in female patients over the age of 50 that posterior multifocal choroiditis may be more common in women over age of 50 [54] and that chest X-ray is often not sufficient to identify associated pulmonary sarcoidosis. In one study, 11 of 17 patients with a negative chest X-ray had findings suggestive of sarcoidosis on chest computed tomography (CT) [55]. This finding has been supported by others [54] and has led to the recommendation that women over 50 years of age with a clinical presentation consistent with ocular sarcoidosis should undergo chest imaging with CT rather than chest X-ray.

## 4. Differences in Treatment

Patients with sarcoidosis have been successfully treated with numerous anti-inflammatory agents, ranging from topical and systemic corticosteroids to antimetabolites and monoclonal antibodies. Because sarcoidosis often occurs in patients of childbearing age, special considerations must be made in patients who may have planned or unplanned pregnancy. The pregnancy class of many medications used to treat sarcoidosis is listed in Table 1. Several of the systemic agents used to treat sarcoidosis are contraindicated during pregnancy.

A recent international poll of sarcoidosis specialists found that 80% of physicians considered MTX their preferred second-line treatment option for sarcoidosis, after systemic corticosteroids [56]. They also reported that, in cases of ocular sarcoidosis refractory to topical corticosteroids, MTX is their preferred first-line drug rather than systemic corticosteroids [56]. Patients should be adequately educated on the risk of birth defects associated with use of MTX and provided with information on appropriate birth control. Although women who plan to get pregnant are at a greater risk of causing direct harm to their unborn fetus, experts agree that both men and women should stop the medication at least 3 months prior to any planned pregnancy [56].

Topical and periocular corticosteroids are commonly used to treat uveitis during pregnancy. In the case of topical administration, patients should be educated on punctual occlusion after administering the medication to reduce systemic absorption. Intravitreal bevacizumab, a class C drug, has reportedly been used for choroidal neovascularization, a potential complication of posterior segment inflammation which can lead to significant and permanent vision loss, in pregnant patients. In a small series of 4 patients treated off-label with intraocular bevacizumab, vision improved and no adverse events were observed [57]. The use of medications during pregnancy should be individualized and must include discussions regarding the risks and benefits of treatment.

## 5. Differences in Prognosis

Patients with sarcoidosis often report a diminished health-related quality of life [58], with a range of symptoms such as emotional distress, lung problems, pain, physical limitations, fatigue, social limitations, eye issues, skin disorders, and sleep disruption [59]. Side effects of treatments for sarcoidosis may also negatively impact quality of life for these patients [60]. Patients with ocular sarcoidosis have a significantly diminished quality of life based on a visual questionnaire when compared to patients without ocular disease. This was particularly true in patients with vision of 20/100 or worse [61]. A prospective, cross-sectional survey study found that women with sarcoidosis showed a greater degree of functional impairment than men, particularly with regard to physical health [62]. There was no difference found in emotional and daily quality of life in men versus women [63].

Extrapulmonary manifestations of sarcoidosis differ by race and sex. Black Americans have a higher prevalence of extrathoracic involvement [30]. A recent study demonstrated that female smokers were more likely to have a reduced diffusion capacity for carbon monoxide than male smokers, and both female sex and smoking are associated with the development of extrapulmonary manifestations of the disease [64].

Death from sarcoidosis is closely associated with respiratory, cardiac, neurologic, and hepatic involvement [4]. A recent report highlighted an increased rate of mortality in recent years, specifically citing a 30% increase in men and 50% increase in women in 2007 compared to 1988 [63]. Although sarcoidosis deaths were most common in younger patients (35–44 years), the increase in mortality was greatest in older individuals (55–74 years), specifically among non-Hispanic black females. The cause of death was determined to be sarcoidosis in almost 60% of patients, while other causes included ischemic heart disease, cardiomyopathy, lung cancer, and pneumonia [65].

## 6. Proposed Reasons for Sex Differences

Women tend to have higher rates of many autoimmune diseases [66], including sarcoidosis. In fact, autoimmune disease is the fifth leading cause of death among women [66]. Proposed reasons for sex differences in incidence of autoimmune disease have included environmental factors such as hormonal and genetic effects, gender-biased activities, occupational exposure, medications, smoking, and vitamin D deficiency in women [67]. Women may be more likely to develop autoimmune disease due to hormonal variation during menstruation, pregnancy, and childbirth. The natural breakdown of epithelial tissue that occurs during these events may increase a woman's exposure to triggers of autoimmune inflammation [68, 69]. In the case of sarcoidosis, this may be of particular importance, as a 2011 study into risk of sarcoidosis among adults exposed to the World Trade Center (WTC) catastrophe found that firefighters, police officers, lower Manhattan residents, area workers, and even passers-by were found to have an increased risk of sarcoidosis. This study specifically concluded that working on the WTC debris pile was associated with an increased rate of post-9/11 sarcoidosis diagnosis [70].

Gender differences may also be related to genetics. A recent Brazilian study found that certain HLA alleles were more likely to be found in mestizos and blacks with sarcoidosis [71]. It is possible that men and women carry these particular haplotypes to varying degrees. Microchimerism and epigenetics have also been implicated in the reason behind female predominance in many autoimmune diseases, including sarcoidosis [72].

## 7. Conclusions

Almost every aspect of sarcoidosis, from epidemiology and clinical presentation to treatment options and prognosis, is influenced by patient sex. Men are diagnosed at a younger age than women, and the onset of sarcoidosis in women follows a bimodal distribution with a second peak of onset occurring after age of 50. In this older cohort of patients, chest X-ray may not be sensitive enough to identify pulmonary changes and computed tomography is recommended. Women tend to have more CME and a worse overall visual prognosis than men. Women are more likely to have cutaneous involvement than men, and the mortality rate is higher in women. Several medications used to treat sarcoidosis are contraindicated in pregnancy; therefore, discussions regarding birth control should be thorough and repeated at every visit.

## Figures and Tables

**Table 1:** Pregnancy classes of medications used to treat systemic sarcoidosis.

Category	Systemic medication	Pregnancy class	
Corticosteroid	Prednisone	C	Risk cannot be ruled out
	Methylprednisolone	C	Risk cannot be ruled out

Antimetabolite	Methotrexate	X	Should not be used during pregnancy
	Mycophenolate	D	Positive evidence of risk
	Azathioprine	D	Positive evidence of risk

Calcineurin inhibitor	Cyclosporine	C	Risk cannot be ruled out

Monoclonal antibody	Infliximab	B	No evidence of risk in humans
	Adalimumab	B	No evidence of risk in humans
	Etanercept	B	No evidence of risk in humans

Alkylating agents	Chlorambucil	D	Positive evidence of risk
	Cyclophosphamide	D	Positive evidence of risk
